# A case report of systemic lupus erythematosus complicating interstitial lung disease and thickened pericardium treated with tofacitinib

**DOI:** 10.1097/MD.0000000000039129

**Published:** 2024-07-26

**Authors:** Xiaoying Li, Kaoye Luo, Dandan Yang, Chunfeng Hou

**Affiliations:** a Department of Rheumatology, Baise People's Hospital, Baise, China; b Department of Radiology, Baise People's Hospital, Baise, China; c Department of Rheumatology, Baise People's Hospital, Jining, China.

**Keywords:** case report, interstitial lung disease, systemic lupus erythematosus, thickened pericardium, tofacitinib

## Abstract

**Rationale:**

Systemic lupus erythematosus (SLE) is a chronic autoimmune disease that damages multiple organs and systems, including the lungs, kidneys, and heart. The respiratory system is commonly affected by SLE, leading to problems such as pleurisy, pleural effusion, and interstitial lung disease (ILD). In addition, SLE can involve the heart, with pericarditis being the most common manifestation. Notably, pericardial effusion frequently accompanies pericarditis involved by SLE, and aspects such as thickened pericardium (TP) can be challenging to detect early on. There are limited reports on TP and even fewer reports on the treatment of ILD with TP. This study investigates the clinical treatment of SLE complicating ILD and TP and reports on a successful case treated with tofacitinib, offering new strategies for managing such patients.

**Patient concerns:**

A 35-year-old female patient presented to the hospital with polyarticular swelling and pain that had been ongoing for over 4 years, as well as recurrent chest pain for 2 years that worsened over the course of 1 day.

**Diagnoses:**

The patient was diagnosed with SLE complicating ILD and TP, with hematologic involvement.

**Interventions:**

Treatment involved the administration of tofacitinib in combination with low-dose methylprednisolone (MP) and mycophenolate mofetil (MMF).

**Outcomes:**

The patient experienced recurrent chest pain and difficulty in reducing glucocorticoids (GCs), but the patient conditions were improved upon the addition of tofacitinib. The patient has been followed up for 16 months, and the patient MP dosage has been reduced to 6 mg once daily. The patient condition remains stable without recurrence, and the patient quality of life has improved.

**Lessons:**

In cases of SLE complicating ILD and TP, when tapering GCs is difficult, treatment with tofacitinib can be effective in achieving remission and maintaining stability.

## 1. Introduction

Systemic lupus erythematosus (SLE) is a connective tissue disease that is systemic and inflammatory, mediated by autoimmunity.^[1]^ Among autoimmune diseases, SLE is the most common one affecting the respiratory system.^[2]^ The pulmonary manifestations of SLE encompass various conditions, including airway diseases, pleurisy, interstitial lung disease (ILD), alveolar hemorrhage, acute lupus nephritis (LN), pulmonary hypertension, shrinking-lung syndrome, and thromboembolic diseases.^[3,4]^ Among these manifestations, pleurisy is the most prevalent in SLE, affecting approximately 60% of patients.^[4,5]^ SLE-associated ILD is relatively rare, affecting around 1% to 15% of patients.^[6]^ Lung involvement serves as a significant prognostic indicator, as it is associated with a twofold or greater increase in mortality. Moreover, it negatively affects patient-reported and patient-presented outcomes.^[7]^ On the other hand, the incidence of cardiac involvement in SLE exceeds 50%. It may impact the pericardium, myocardium, endocardium, coronary arteries, and conduction system. Pericarditis is the most common, followed by myocarditis, coronary atherosclerosis, and valvulopathy.^[1]^ Cardiac involvement is the leading cause of morbidity and poor prognosis in SLE patients, and its clinical manifestations can range from asymptomatic to atypical in presentation.^[1,8]^

Over the past 50 years, the treatment of SLE has relied heavily on long-term nonspecific immunosuppressive drugs, which often lead to complications. In response, a treat-to-target (T2T) method for SLE was proposed.^[9]^ The goals of SLE treatment include achieving remission, preventing relapse and organ damage, and minimizing drug toxicity. The T2T strategy recommends using the lowest possible dose of glucocorticoids (GCs) for disease control, and if feasible, discontinuing GCs altogether. However, in the case presented in this study, the patient experienced recurrent chest pain, making it difficult to taper the GCs and even more challenging to discontinue them. Therefore, we proposed a targeted therapy to address the patient immune abnormalities.

The pathogenesis of SLE involves various abnormalities in the acquired and innate immune system mediated by cytokines. When cytokines and cell surface molecules bind to receptors, they initiate a series of intracellular signaling processes that result in changes in cellular function. Enzymes that phosphorylate signaling molecules in cell signaling are called kinases. Janus kinase (JAK) is an intracellular tyrosine kinase that binds to receptors and serves as an important signaling intermediary for a range of cytokines and hormones. There are 4 types of JAK family tyrosine kinases (JAK1, JAK2, JAK3, and TYK2), and there are over 50 cytokines, such as IL-2, 3, 4, 5, 6, 12, and interferons IFNs, that act through specific combinations of JAKs.^[10–15]^ JAK inhibitors (JAKis) interfere with multiple cytokine signaling pathways and play an immunomodulatory role in various pathological processes. Among JAK inhibitors, tofacitinib stands out as an oral targeted synthetic antirheumatic agent that primarily inhibits JAK1 and 3, thereby blocking the cytokine signaling pathway and preventing the recruitment of leukocytes, as well as the activation and expression of pro-inflammatory cytokines at sites of inflammation.

This case report presents a patient with SLE, accompanied by ILD and TP. After receiving tofacitinib treatment, the patient condition stabilized, and the use of GCs was successfully reduced. This provides a new idea and method for treating similar patients.

## 2. Case report

The patient was a 35-year-old female who initially presented to the rheumatology department in our hospital in September 2017. The patient complained of swelling and pain in the metacarpophalangeal joints of both hands. Over time, these symptoms extended to involve the joints of both knees and ankles. Auxiliary examinations revealed leukocyte and neutrophil levels of 2.7 × 10^9^/L and 1.08 × 10^9^/L, respectively. Other test results revealed an antinuclear antibodies (ANA) titer of 1:1000, positive anti-SSA antibodies, and positive anti-double stranded DNA antibodies, while the rest of the test results were negative. Complement_4_ levels were measured at 0.12 g/L, while IgG levels stood at 21.41 g/L. Based on these findings, the patient was diagnosed with “hematologic involvement of SLE and leukopenia.” The patient was prescribed 30 mg of prednisone orally on a daily basis. After 2 weeks, the patient experienced a significant decrease in arthralgias, and the blood test results returned to normal. The dosage of prednisone was gradually reduced, and the patient was also prescribed 10 mg of methotrexate once a week and 200 mg twice daily (BID) of hydroxychloroquine (HCQ). The patient condition stabilized, and the dosage of prednisone was gradually reduced to 10 mg every other day. However, the patient did not follow-up regularly and discontinued the patient medication on the patient own for more than 8 months.

**Figure 1.**
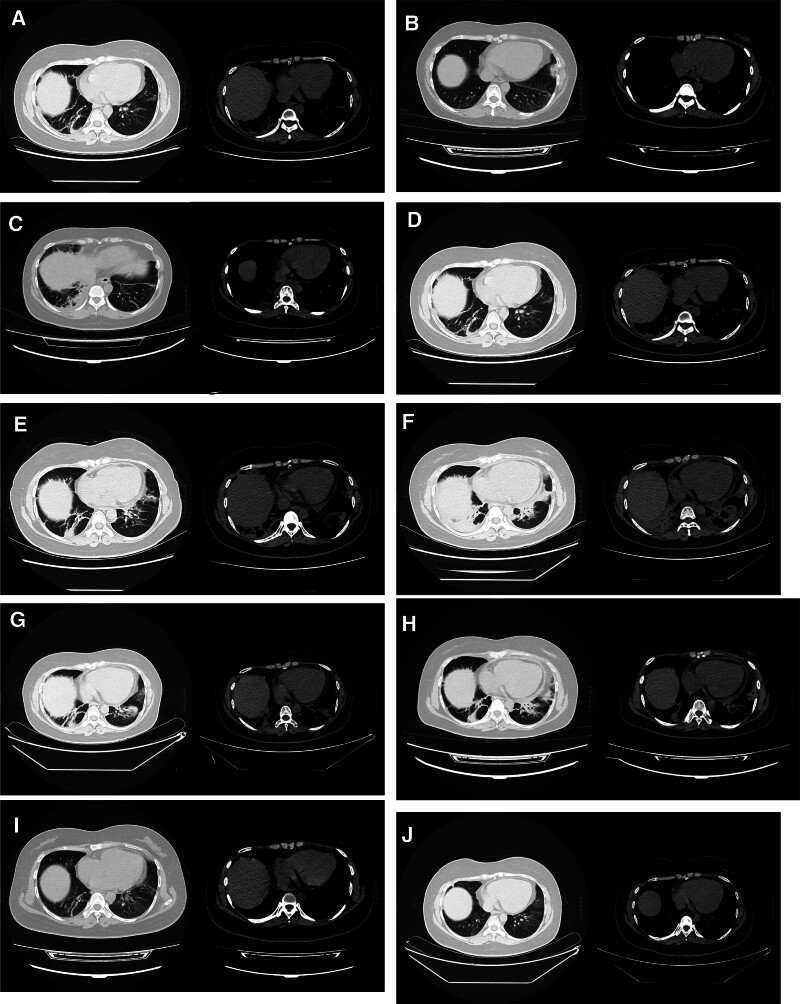
CT images of the lungs and mediastinal windows of patients at different time points. (A) Multiple patchy, streaky, dense shadows were observed in both lungs, especially in the inferior lobe. Local mild pericardial thickening, approximately 3 mm thick, was also observed. (B) 40 mg of MP was administered; both lung lesions were significantly more resorbed than before, and the TP improved. (C) MP was reduced to 6 mg QD. In addition, it was observed that the inferior lobe lesion in the right lung had progressed further compared to previous assessments, demonstrating pleural thickening. (D) MP was increased to 20 mg QD; the inferior lobe lesion of the right lung was more resorbed compared to previous assessments. (E) MP was reduced to 8 mg QD; inflammation in the inferior lobe of both lungs had progressed further compared to previous assessments. ILD was observed in the right lung, and a newly identified TP of approximately 9 mm was observed. (F) The MP dosage was not increased. The inflammation in the inferior lobes in both lungs was aggravated following anti-infective treatment compared to previous assessments, with a TP of approximately 12 mm. (G) MP was increased to 24 mg QD; the inflammation in the inferior lobe in both lungs was reduced compared to the previous resorption findings, and the TP had significantly reduced. (H) MP was reduced to 8 mg QD. Additionally, it was noted that the lesions in the inferior lobes of both lungs had increased compared to previous assessments. Furthermore, a new pleural thickening of approximately 11 mm was observed. (I) MP was not increased, and tofacitinib 5 mg BID was added. There was a notable reduction in the resorption of ILD in both lungs and a significant reduction in the TP by approximately 2 mm. (J) MP at 6 mg QD and tofacitinib at 5 mg BID were administered. There was a slight reduction in exudation due to resorption in both lungs, and no TP was observed. BID = twice daily, CT = computed tomography, ILD = interstitial lung disease, MP = methylprednisolone, TP = thickened pericardium.

On August 12, 2020, the patient was admitted to the hospital with a 1-week history of cough and chest pain. The patient reported coughing up a small amount of white mucous sputum and chest pain that worsened with coughing and deep breathing. Importantly, the patient chest pain was not exacerbated by changes in body position, and the patient did not experience fever. A blood test showed no abnormalities, except for a C-reactive protein (CRP) level of 26.09 mg/L, Complement_4_ level of 0.10 g/L, and IgG level of 26.53 g/L. Furthermore, ANA levels were elevated at 1:320, and anti-SS-A was positive. The electrocardiogram (ECG) was normal, but a chest computed tomography (CT)scan revealed inflammation in both lungs, a small amount of pleural effusion on the left side, bilateral pleural thickening, and mild thickening of the pericardium with a localized thickness of approximately 3 mm (Fig. 1A). Initially, lung infection was suspected, and the patient was treated with “ceftriaxone + cefoperazone-sulbactam.” However, the patient cough and chest pain did not subside. Seven days later, a repeat chest CT scan showed no significant change in the inferior lobe lesions in both lungs. Further bronchoscopy revealed no abnormality in the bronchial tubes, and analysis of the patient alveolar lavage fluid did not detect any specific pathogens. Three-dimensional imaging of the pulmonary artery CT angiography (CTA) + vessel did not show any abnormalities, leading to the consideration of ILD caused by SLE. The patient was given 40 mg once daily (QD) of intravenous methylprednisolone (MP), and the patient chest pain gradually subsided. The patient was discharged from the hospital in good condition and continued taking 40 mg QD of MP tablets and 200 mg BID of HCQ.

A follow-up lung CT imaging on September 15, 2020, showed significant resorption of inflammation in both lungs compared to before treatment, with improvements in TP (Fig. 1B). Subsequently, the dosage of MP was gradually reduced. However, follow-up lung CT imaging 3 months later revealed progression of the inferior lobe lesions in both lungs compared to the previous scan, with increased involvement on the right side. Despite this, the patient did not experience chest tightness, chest pain, cough, or fever. Mycophenolate mofetil (MMF) was added at a dosage of 0.75g BID, and the rate of GC tapering was slowed down. MP was reduced to 6 mg QD, but the patient subsequently presented again with chest and back pain, worsening cough, and deep breathing. The lung CT imaging on July 20, 2021, showed further progression of the inferior lobe lesion in the right lung and resorption of lesions in the left lung, with the presence of a new right pleural effusion (Fig. 1C). A color Doppler flow imaging (CDFI) on the heart showed no abnormalities. The MP dosage was increased to 16 mg QD, resulting in relief from the chest pain.

Follow-up lung CT imaging on August 9, 2021, showed slight improvement in the inferior lobe lesion on the right side and resorption of the right pleural effusion, while the other findings remained generally unchanged (Fig. 1D). Two months later, the MP dosage was reduced to 8 mg QD, and a routine blood test showed leukocytes at 1.7 × 10^9^/L and neutrophils at 0.12 × 10^9^/L. MMF was discontinued, and the bone marrow routine and biopsy showed no significant abnormalities. MP was increased back to 16 mg QD, and the leukocyte count returned to normal. However, after reducing MP to 8 mg QD, multiple repeat blood tests showed a decrease in leukocytes. The patient received intermittent treatment with human granulocyte colony-stimulating factor and leukocyte-boosting drugs, along with 1 mg BID of tacrolimus.

On August 25, 2022, the patient was readmitted to the hospital after experiencing a fever with a peak temperature of 38.3°C 1 day earlier. The patient also complained of severe and intolerable tingling pain in the left thoracic back, which was aggravated by coughing and respiration but alleviated by a flexed position.

The patient was previously in good health.

On examination, the temporal temperature was 38.3°C, the heart rate was 120 beats per minute, the patient appeared tired and uncomfortable because of fever and pain.

No abnormalities were found in the following tests and assays: anti-double-stranded DNA antibodies, anti-neutrophil cytoplasmic antibody, anticardiolipin antibody, anti-β2-GP1, lupus anticoagulant, urinalysis, CK-MB and troponin, B-type natriuretic peptide, hepatitis B surface antigen, hepatitis C antibody, HIV antibody and syphilis spirochete antibody, bacterial endotoxin + fungal dextran, influenza A and B viral nucleic acid, 24-hour urine, Epstein-Barr virus, cytomegalovirus infection, combined IgM antibodies against 9 respiratory pathogens, blood culture, T-spot. TB, and tumor series. Other laboratory test results are shown in Table 1.

**Table 1:** Laboratory date.

Variable	Reference range	During hospitalization
Leukocyte (10^9^ /L)	3.5–9.5	2.0
Neutrophils (10^9^ /L)	1.8–6.3	0.52 × 10^9^/L
Erythrocyte sedimentation rate (mm/h)	0–20	56
CRP (mg/L)	0–6.2	87.58
CH50 (U/mL)	23–46	44
C1_Q_ (mg/dL)	15.7–23.7	18.7
C_4_ (g/L)	0.18–0.4	0.09
C_3_ (g/L)	0.8–1.85	1.02
Interleukin-6 (pg/mL)	0–10	56.1
Procalcitonin (ng/mL)	0–0.005	0.08
ANA	＜1:100	1:3200, anti-SS-A positive (++)
Ferritin (µg/L)	10–291	460.90
Lymphocyte subset assay		
CD3^+^% (%)	65–79	84.23%
CD3-CD56+ (%)	10.04–19.78	0.98%
Absolute CD3 + count (per μL)	955–2860	372
Absolute CD3^+^ CD4^+^ count (per μL)	550–1440	187

ECG showed sinus tachycardia and partial T-wave changes. Chest CT imaging revealed progressive inflammation in both lungs compared to previous images, as well as a small amount of pericardial effusion, pleural thickening on both sides, and a TP of about 9 mm (Fig. 1E). CDFI on the heart showed no abnormalities. Abdominal CT imaging did not show any abnormalities. Coronary CTA + 3D reconstruction indicated no significant abnormality in coronary CTA, a low risk of coronary artery calcification, TP of about 9 mm, and a small pericardial effusion. A follow-up chest CT imaging after 1 week showed further progression of inflammation in both lungs compared to previous images, with thickening of the pleura on both sides, a TP of about 12 mm, and a small amount of pleural effusion on both sides (Fig. 1F). Fibrilloscopy revealed inflammatory bronchial changes. A routine test of alveolar lavage fluid showed no abnormalities, and no acid-fast bacilli were observed on the smear. The cryptococcal antigen test yielded negative results. Fungal culture and identification, including drug sensitivity testing, did not detect *Candida* or other fast-growing fungi. Similarly, the *Pneumocystis jiroveci* nucleic acid test was negative. Bacterial culture and identification, along with drug sensitivity testing, did not isolate common pathogenic bacteria or *Haemophilus*.

Considering the possibility of lung infection in the patient, tacrolimus was discontinued and cefoperazone-sulbactam was administered for anti-infective treatment, but the patient chest pain persisted. Follow-up lung CT imaging revealed that the inflammation was worsening compared to before. Both blood cultures and alveolar lavage fluid pathology showed no abnormalities. The characteristics of the ILD were very similar to the initial episode, and the exacerbation of the lesions occurred after a decrease in the dose of GCs, which supports the idea that SLE is complicating the ILD. The patient declined to undergo a tissue biopsy of the affected tissues. The lung lesions remained the same as before, but the chest pain has turned into a severe stabbing pain, mainly on the left side of the chest and back, and was relieved when the patient was in a flexed position. Lung CT imaging indicated significant thickening of the pericardium, prompting a recommendation for a pericardiocentesis biopsy. However, the patient refused to undergo the biopsy. The tuberculosis-specific enzyme-linked immunospot assay (T-SPOT.TB) and tumor marker tests showed no abnormalities, and there was a change in the ECG results compared to earlier. Therefore, it was concluded that the patient chest pain was a result of pericarditis in SLE. Finally, it was determined that the patient had active SLE with recurrence of ILD and concurrent pericarditis with notable pericardial thickening. As a result, the patient was prescribed 24 mg QD of MP along with 0.5g BID of MMF. The patient experienced relief from chest pain, and the patient temperature returned to normal. A lung CT scan conducted 8 days later indicated a reduction in inflammatory response in both lungs, as well as a significant decrease in TP (Fig. 1G). Consequently, the patient was discharged from the hospital in good condition.

After being discharged from the hospital, the patient continued taking 24 mg QD of MP, 0.5 g BID of MMF, and 200 mg QD of HCQ. The patient condition stabilized, and the dosage of MP was gradually reduced. Three months later, when MP was lowered to 8 mg QD, the patient experienced chest pain again, similar to the previous episode. A follow-up CT scan of the lungs revealed bilateral lung inflammation with a slightly larger lesion in the inferior lobe compared to the previous examination. The transverse process measured approximately 11 mm, with a small amount of pericardial effusion, while the rest of the findings were similar to the previous examination (Fig. 1H). CDFI of the pericardium indicated a TP of approximately 3 mm. Due to cost considerations, the patient received 1 day of 20 g of gamma globulin before discontinuation. Tofacitinib was added to the treatment regimen at a dose of 5 mg BID, while MP was continued at 8 mg QD. One month later, the patient reported no chest pain. A follow-up lung CT scan on January 7, 2023, showed significant improvement in the bilateral pneumonic lesions, with almost complete resolution of the pericardial effusion. The TP had reduced by approximately 2 mm, and the pleural thickening had improved (Fig. 1I). At the time of publication, the patient condition remained stable. A follow-up lung CT scan in February 2024 revealed further improvement, with decreased exudates in both lungs and no pericardial thickening (Fig. 1J). Currently, the patient is taking 6 mg QD of MP.

## 3. Discussion

SLE is a systemic autoimmune disease characterized by abnormal immune system activity.^[16]^ It presents with a wide range of clinical manifestations, including respiratory, cardiovascular, renal, cutaneous, and neuropsychiatric symptoms.^[17]^ The pulmonary manifestations of SLE include parenchymal lung disease (such as ILD and acute pneumonia), pleural involvement (resulting in pleurisy and pleural effusion), and pulmonary vascular complications (including pulmonary arterial hypertension, pulmonary embolic disease, and pulmonary vasculitis). The exact prevalence of lung disease associated with SLE is uncertain, with previous studies reporting widely varying rates; however, recent research suggests a prevalence of 50% to 70%.^[18–20]^ In particular, SLE-associated interstitial lung disease is estimated to occur in 3% to 9% of cases.^[21,22]^ These pulmonary manifestations have a significant negative impact on patients’ health and physical functioning and are associated with higher mortality rates.^[7]^ The cardiovascular system is a target organ in SLE, affecting the heart, including the pericardium, myocardium, heart valves, and coronary arteries. However, most patients do not display clinical symptoms. The pathogenesis of cardiac involvement in SLE is characterized by a complex series of systemic and local autoimmune processes. This includes the deposition of immune complexes in the vascular wall and perivascular tissues, complement system activation, and infiltration of inflammatory cells, resulting in inflammatory reactions and vascular abnormalities.^[1,8,23]^

Pericarditis is a well-studied cardiovascular manifestation of SLE. It is most prevalent in the early stages of SLE or during recurrences, suggesting that pericarditis, especially pericardial effusion, is associated with SLE activity and serves as a marker for SLE activity. As a result, it has been included in the American College of Rheumatology (ACR) classification criteria for SLE. Pericarditis in SLE is characterized by focal or diffuse fibropericarditis with or without exudation. Pericardial effusion, detected by CDFI, occurs in 11% to 54% of cases.^[1]^ Autopsies reveal pericardial involvement in 62% of SLE patients.^[24]^ Signs and symptoms of acute pericarditis include typical precordial or substernal pain, sometimes accompanied by dyspnea. Fever, tachycardia, and disminished heart sounds may also be present. Additionally, while pericardial friction sounds may be heard, they are rare. Approximately 25% of SLE patients develop symptomatic pericarditis during the patient disease, and asymptomatic pericardial effusion occurs in 40% of cases.^[1]^ Moreover, only 2 to 4% of SLE patients present with pericarditis or pericardial compression signs as the initial manifestation. Mild pericarditis can be effectively treated with non-steroidal anti-inflammatory drugs and GCs. However, if massive pericardial effusion occurs, large doses of GCs, including high-dose GC therapy, or a combination with immunosuppressive agents such as cyclophosphamide or MMF, may be necessary. In addition, immunosuppressive agents such as methotrexate, azathioprine, MMF, and intravenous immunoglobulin may provide benefits for recurrent pericarditis. Although there is no conclusive evidence regarding the efficacy of biologics in treating pericarditis in SLE, drugs such as rituximab or belimumab may be effective in some patients.

Current research on pericarditis in SLE has primarily focused on pericardial effusion, with less attention and fewer reports on TP, along with limited discussions on TP treatment. In this case, the patient experienced recurrent chest pain while attempting to taper off GCs, making further reduction challenging. When the patient experienced chest pain, a chest CT scan revealed ILD with significant pericardial thickening, reaching up to 12 mm in its thickest section. Interestingly, there was little to no pericardial effusion indicated by CDFI of the heart. Moreover, despite the absence of a definitive cause for pericardiocentesis, laboratory and imaging tests ruled out infection (such as tuberculosis) or tumor. The response to treatment also did not support the presence of infection or tumor. Therefore, we concluded that the patient had pericarditis due to SLE.

Treating TP can be highly challenging due to the absence of standardized guidelines. Typically, appropriate disease-modifying antirheumatic drugs (DMARDs) are chosen based on the severity of pericarditis and whether other organs are involved. In this study, the patient had comorbid ILD. According to Chinese guidelines for the diagnosis and treatment of connective tissue disease-associated interstitial pneumonia, the first-line therapy typically includes GCs, cyclophosphamide, and MMF. However, because the patient was a young woman with reproductive needs who refused cyclophosphamide, GCs and MMF were prescribed instead. The patient condition improved initially, but when the dose of MP was reduced to 2 tablets QD, the patient experienced recurrent episodes of chest pain accompanied by leukopenia. These episodes led to multiple hospitalizations and severely impacted the patient quality of life. Belimumab injections were recommended by the medical team, but the patient refused, creating a treatment dilemma. Further research revealed that SLE is a recognized risk factor for early cardiovascular disease due to dysregulation of immune pathways. About half of SLE patients have elevated levels of type I interferons in the patient blood, which can enhance the risk of cardiovascular disease when interacting abnormally with neutrophils.

The JAK/STAT signaling pathway plays a role in SLE pathogenesis, especially in conjunction with interferons,^[25]^ making it an attractive target for therapy. JAKis inhibit various cytokine signaling pathways and modulate immunity and inflammation, providing a multi-targeted approach to suppressing immunopathogenesis in autoimmune diseases. Multiple clinical trials for SLE treatment using JAKis are currently underway, each focusing on different selectivities for JAK family proteins. Previous utilization of JAKis in SLE was grounded in in vitro experiments and studies conducted on mice.^[26]^ Animal studies have shown that tofacitinib, a JAKi, significantly improves clinical manifestations, immune dysregulation statuses, and vascular dysfunction in SLE animal models.^[27]^ In addition, a study by Hasni et al demonstrated that short-term administration of tofacitinib has a good safety profile and improves cardiometabolic and immunologic parameters associated with premature atherosclerosis in SLE patients with mild to moderate disease activity.^[28]^ More recently, tofacitinib has shown efficacy in treating joint symptoms, rash, recalcitrant alopecia, and LN caused by SLE, as well as interstitial lung diseases associated with antisynthetase syndrome and anti-melanoma differentiation-associated gene 5 antibody-positive dermatomyositis.^[29,30]^ Based on the underlying mechanisms of SLE, the mode of action of JAKis, and previous successful cases of treating SLE with tofacitinib, we hypothesized that tofacitinib could be effective in treating SLE complicated by ILD and TP. Therefore, we added 5 mg BID of tofacitinib to the patient treatment regimen, in combination with MMF, without increasing the dosage of MP. The patient experienced relief from chest pain after 1 month. At the time of follow-up, the patient condition remained stable, and a follow-up CT scan showed signs of inflammation resorption in the lungs. There were no tofacitinib-related adverse events, and MP has been successfully reduced to 6 mg once daily.

## 4. Conclusion

In conclusion, this study reports the first application of tofacitinib in treating SLE complicated by ILD and TP. Tofacitinib not only improved SLE with ILD and TP but also reduced the GC dosage. However, it is important to note that this study is based on a single case report. Therefore, it is crucial to collect more clinical data by expanding the number of medical records in the future and extending the follow-up period to ensure long-term efficacy and safety observations.

## Author contributions

**Conceptualization:** Xiaoying Li, Chunfeng Hou.

**Data curation:** Xiaoying Li, Kaoye Luo, Dandan Yang.

**Supervision:** Chunfeng Hou.

**Writing – original draft:** Xiaoying Li.

**Writing – review & editing:** Chunfeng Hou.
